# Editorial: The use of metabolic engineering techniques to increase the productivity of primary and secondary metabolites within filamentous fungi

**DOI:** 10.3389/ffunb.2023.1178290

**Published:** 2023-04-21

**Authors:** Koichi Tamano, Daren W. Brown, Akira Yoshimi

**Affiliations:** ^1^ Bioproduction Research Institute, National Institute of Advanced Industrial Science and Technology (AIST), Sapporo, Japan; ^2^ Computational Bio Big-Data Open Innovation Laboratory (CBBD-OIL), National Institute of Advanced Industrial Science and Technology (AIST), Tokyo, Japan; ^3^ Mycotoxin Prevention and Applied Microbiology Research (MPM), National Center for Agricultural Utilization Research (NCAUR), Agricultural Research Service, USDA, Peoria, IL, United States; ^4^ Laboratory of Environmental Interface Technology of Filamentous Fungi, Graduate School of Agriculture, Kyoto University, Kyoto, Japan; ^5^ Laboratory of Terrestrial Microbial Ecology, Graduate School of Agriculture, Kyoto University, Kyoto, Japan

**Keywords:** filamentous fungi, valuable metabolite, metabolic engineering, production enhancement, gene knockout, gene overexpression

Filamentous fungi are multicellular eukaryotic microorganisms that absorb nutrients from their environment. The hydrolytic enzymes secreted for this osmotrophic lifestyle have been greatly exploited by a variety of industries. Many filamentous fungi today serve in mass production of high-value enzymes such as amylases, proteases, and lipases, as well as high-value chemicals for the food, chemical, and pharmaceutical industries. Interest in filamentous fungi was sparked in 1917 by *Aspergillus niger*, which was discovered to produce large amounts of citrate, a primary metabolite mainly used today as a food additive (Cairns et al., 2018). In 1928, *Penicillium rubens* was found to produce penicillin, a secondary metabolite with antibacterial properties (Fleming, 1929). Subsequent work ushered in the antibiotic revolution, which has saved millions of lives. Research on diverse filamentous fungi has identified many other metabolites, of which some are widely used today as pharmaceutical agents (e.g., cyclosporin, lovastatin), pigments (e.g., carotenoids), platform chemicals (e.g., itaconic acid), and cosmetics (e.g., kojic acid).

In the wider field of microbial production of valuable compounds, bacteria (e.g., *Escherichia coli*, *Streptomyces* sp., *Corynebacterium* sp.) and yeasts (e.g., *Saccharomyces cerevisiae*, *Pichia pastoris*, *Yarrowia lipolytica*) are commonly utilized as production hosts. However, when the genomes of the first filamentous fungi were sequenced two decades ago, researchers realized that they contained more genes involved in degradation of plant polymers (i.e., polysaccharides, protein) and production of secondary metabolites than had been previously realized. Nevertheless, the challenges of realizing this potential are substantial: filamentous fungi are difficult to grow at high density in liquid cultures due to pellet formation; genetic manipulation methods are complicated, as compared to the cases of bacteria and yeasts; and strain development via recombination is time-prohibitive. Therefore, the implementation of existing strategies in new ways and the development of new metabolic and genetic engineering techniques is essential for the production of high-value enzymes and primary and secondary metabolites from fungi for scientific and commercial use.

This Research Topic is dedicated to breakthrough metabolic engineering techniques and strategies for the production of primary and secondary metabolites by filamentous fungi.

Dao et al. elegantly improved the filamentous fungus *Aspergillus oryzae* to enable it to serve as a platform organism for the heterologous production of metabolites by eliminating synthesis of the secondary metabolite kojic acid in strain NSAR1. As a candidate host for chemical and enzyme production, *A. oryzae* was already well known for producing a limited variety and low quantity of secondary metabolites. Among them, kojic acid is the most abundant. In this study, the gene *kojA*, responsible for kojic acid biosynthesis, was deleted in NSAR1. In addition to not producing kojic acid, the mutant produced little other secondary metabolite (i.e., it was found to be a secondary metabolite non-producing strain). With the significant reduction in production of extraneous chemicals, the efficiency and quantity of directed metabolite synthesis via heterologous expression by this host was greatly increased.

Yamaguchi et al. characterized a biosynthetic gene cluster (BGC) involved in synthesis of the novel cyclic peptide compound KK-1 in the filamentous fungus *Curvularia clavata*. They identified candidate BGCs using two methods. The first method was to sequence the genome of the fungus and mine genes with non-ribosomal peptide synthetase (NRPS) motifs. The second method was to identify BGCs whose expression was induced after growth in target-compound-producing culture conditions. Both methods led to the same candidate: a 10-gene BGC spanning 71 kb. Deletion mutants of cluster biosynthetic genes eliminated KK-1 production, while overexpression of the cluster transcription factor gene enhanced KK-1 production. The series of experiments described represents an excellent laboratory roadmap leading to the identification and characterization of a novel BGC.

In their opinion article, Tamano and Yoshimi summarized and commented on research reports relevant to valuable metabolite production in filamentous fungi. They described methods that can be used to improve the metabolism of filamentous fungal strains to make them more suitable for mass production of target metabolites. In addition, heterologous production was cited as an important method for mass production in cases where metabolic modification cannot be applied to the original producing strains, owing to fermentation difficulties or to intractable genetic methods.

Umemura and Tamano summarized efforts to improve the production of peptidyl secondary metabolites. Fungal peptidyl compounds are synthesized by either an NRPS or a ribosome. Proteins synthesized by ribosomes that undergo post-translational modifications are referred to as RiPPs (i.e., ribosomally synthesized and post-translationally modified peptides). The authors described multiple approaches to improve the production of this important class of secondary metabolites, focusing primarily on directed strain enhancement techniques, and discussed published examples from the literature.

The four papers published in this Research Topic describe an array of technologies and approaches directed at increasing the production of primary and secondary metabolites, including peptidyl compounds, through the improvement of host strains, BGC identification, and implementation of new productivity-enhancing strategies. However, neither efforts to enhance production of polyketide compounds nor the impact of hyphae morphology on production are mentioned. These two factors are important to consider with reference to the production of valuable metabolites. Here, we mention two recent papers that cover these topics. Along with peptidyl compounds, polyketides are a major class of secondary metabolites that are often produced at a low level or not at all in laboratory cultures. Production of the polyketide curcumin was recently increased more than sixfold in *A. oryzae* by deletion of the SREBP regulatory system and by increasing the availability of the polyketide precursor acetyl-CoA by overexpressing *snf1* (Kan et al., 2019). Regarding fungal morphology and its importance in product synthesis in fermentations, even though high-density hyphal liquid cultures are associated with high yields, filamentous fungi often grow in a pelleted form, leading to low yields. Miyazawa et al. (2019) succeeded in dispersing the hyphae of *A. oryzae* by disrupting the gene *agsB*, which is responsible for the biosynthesis of α-1,3-glucan, a major cell wall component, and the genes *sphZ* and *ugeZ*, which are responsible for the biosynthesis of galactosaminogalactan, an extracellular polysaccharide. A mutant strain with these genetic changes produced a significant increase in yields of recombinant protein per unit culture volume. The work described here helps with efforts to increase the production of valuable metabolites in filamentous fungi.

## Author contributions

KT drafted the text of the editorial. DB and AY reviewed and revised the draft. All authors contributed to the article and approved the submitted version.
